# Cost-effectiveness of statin-based primary prevention of cardiovascular disease with electronic pillboxes for people with HIV in China

**DOI:** 10.1186/s12913-026-15173-y

**Published:** 2026-08-04

**Authors:** Yuxuan Li, Wanqing Huang, Lili Cui, Dongmei Zhong, Dong Roman Xu, Hongwei Zhou, Rui Zhao

**Affiliations:** 1 China-Australia Joint Research Center for Infectious Diseases, School of Public Health, Xi’an Jiaotong University Health Science Center, Xi’an, Shaanxi 710061 China; 2 Acacia Lab for Implementation Science, School of Health Management, Southern Medical University, 1023-1063, South Shatai Road, Guangzhou, Guangdong 510515 China; 3 West China Hospital Endocrinology and Metabolism Department, West China School of Nursing, Sichuan University, Chengdu, 610041 China; 4 Dermatology Hospital, Southern Medical University, Guangzhou, China; 5 Center for World Health Organization Studies, School of Health Management, Southern Medical University, Guangzhou, Guangdong China; 6 Microbiome Medicine Center, Department of Laboratory Medicine, Zhujiang Hospital, Southern Medical University, Guangzhou, China; 7 Department of Obstetrics and Gynaecology, Shenzhen Hospital, Southern Medical University, Shenzhen, China; 8 Guangdong Provincial Clinical Research Center for Laboratory Medicine, Guangzhou, China; 9 State Key Laboratory of Multi-organ Injury Prevention and Treatment, Guangzhou, China; 10 Shenzhen Hospital of Southern Medical University, 1333 Xinhu Road, Bao’an District, Shenzhen, Guangdong 518000 China; 11 Microbiome Medicine Center, Department of Laboratory Medicine, Zhujiang Hospital, Southern Medical University, 1333 Xinhu Road, Bao’an District, Shenzhen, 518000 China

**Keywords:** Statins, Cardiovascular disease, HIV, Digital health, Cost-effectiveness

## Abstract

**Objectives:**

Cardiovascular disease (CVD) is the leading non-AIDS cause of death among people with HIV (PWH). Although statins reduce CVD risk by over one-third, poor adherence limits real-world effectiveness. Electronic pillboxes may bridge this gap, but their cost-effectiveness for CVD primary prevention in PWH remains unclear.

**Methods:**

We developed a Markov cohort model simulating 100,000 Chinese PWH (aged 40–75 years) over a lifetime horizon from a healthcare system perspective. Strategies varied by CVD screening frequency (every 6/12/24 months), lipid-lowering regimens (pitavastatin alone, or combined with ezetimibe or PCSK9 inhibitor), and electronic pillbox adherence support for high-risk individuals. Incremental cost-effectiveness ratios (ICERs) were calculated as cost per quality-adjusted life-year (QALY) gained versus status quo at willingness-to-pay thresholds of 1 × and 3 × GDP per capita ($13,445 and $40,335) in China.

**Results:**

Risk-stratified screening every 24 months with pitavastatin alone (without pillboxes) was optimal at 1×GDP per capita (ICER $8,269/QALY), averting 4,298 major adverse cardiovascular events (MACE) and gaining 28,570 QALYs versus status quo. At 3×GDP per capita, 6-monthly screening with risk-tailored therapy (pitavastatin ± ezetimibe) plus electronic pillboxes was optimal (ICER $16,799/QALY), averting 10,169 MACE and gaining 59,125 QALYs. Statin adherence and ezetimibe cost were key drivers of cost-effectiveness.

**Conclusions:**

Risk-stratified CVD prevention integrating periodic screening and tailored pharmacotherapy is cost-effective for PWH in China. Electronic pillboxes become cost-effective when greater healthcare investment is available, supporting a threshold-dependent approach to translating statin efficacy into population-level impact.

**Supplementary Information:**

The online version contains supplementary material available at https://doi.org/10.1186/s12913-026-15173-y.

## Introduction

With the widespread use of antiretroviral therapy (ART), the life expectancy of people with HIV (PWH) has approached that of the general population [1, 2]. However, long-term ART and persistent immune dysfunction also significantly increased cardiovascular disease (CVD) risk in PWH [3–5]. A global meta-analysis reported up to a two-fold higher CVD risk in PWH [6], with myocardial infarction risk increased by 20%–100% compared with people without HIV [7]. In China, CVD has become the leading cause of death among both the general population and PWH, accounting for nearly half of all deaths nationwide [8], and 26.1% of non-AIDS related deaths among PWH [9]. Given the paradigm shift from AIDS-related mortality to non-communicable diseases in PWH, innovative primary prevention strategies are urgently needed, particularly in resource-limited settings [10, 11].

Large-scale studies have confirmed that statins as cholesterol-lowering medications can effectively reduce CVD risk. The 2023 REPRIEVE trial revealed that statins (such as pravastatin) were able to reduce the high risk of CVD in PWH by more than one-third [12, 13]. Another real-world study based on the largest HIV-infected cohort in North America (NA-ACCORD) also indicated that the risk of MACE decreased by 21% among statin initiators [14]. In 2025, guidelines from the U.S. Department of Health and Human Services and the British HIV Association recommended statins for CVD prevention in PWH aged ≥ 40 [15, 16]. The Global Heart Initiative further suggested integrating statins into existing HEARTS hypertension and diabetes treatment plans to expand coverage in LMICs [17]. Beyond statin monotherapy, combination with ezetimibe or PCSK9 inhibitors has been recommended for enhanced efficacy and improved drug–drug interaction safety with ART [18, 19]. To ensure timely identification of high-risk individuals who would benefit from preventative interventions, international guidelines also emphasize the necessity of regular cardiovascular risk assessment [20, 21]. However, the optimal frequency of screening may need to be individualized based on baseline risk, with more frequent monitoring (at least annually) recommended for those with substantial risk for CVD [22].

Despite strong evidence and recommendations, suboptimal uptake and adherence to statins have led to a sustained implementation gap, markedly reducing their real-world benefit. Poor adherence not only increases CVD risk and healthcare costs but also dilutes the population-level benefits of statin therapy. This concern is particularly salient in China, where less than 20% of eligible individuals receive statin therapy despite a large population and high CVD burden [23]. Global adherence rates range from 17.8% to 79.2%, with fewer than half adherent at one year [24, 25]. Digital health technologies, such as electronic pillboxes, provide real-time dosing data and dedicated communication channels, offering an opportunity to bridge this implementation gap. Multiple RCT studies have confirmed the effectiveness of electronic pillbox and digital medication system in promoting medication adherence in tuberculosis and severe mental disorders [26, 27], also suggesting their potential applicability to supporting combination drug regimens among PWH.

While several modelling studies have evaluated the health and economic impact of statins for CVD prevention [28–30], key gaps remain regarding context-specific risk stratification, the value of integrating statins with adherence-enhancing digital interventions, and the feasibility of implementation within China’s health system. To address these gaps, we conducted a cost-effectiveness analysis evaluating the economic and health benefits of integrated statins and electronic pillbox use among PWH in China, providing a health economics basis for policymakers planning large-scale statins rollout.

## Methods

### Modelling

We developed a Markov model comprising 24 health states to simulate the progression of CVD among PWH in China, from initial susceptible status with different CVD risk strata, to first-onset CVD events, recurrent CVD and death. The model aimed to evaluate the cost-effectiveness of different statin-based primary prevention strategies for CVD in combination with other cholesterol-lowering drugs, various frequency for CVD risk screening, and electronic pillbox use as an adherence-enhancement tool. We simulated a static cohort of 100,000 individuals living with HIV by using a monthly cycle length over a lifetime horizon, covering the age range from 40 to 75 years. The model incorporated several key health states, including PWH at risk for CVD, PWH with first/recurrent CVD, and death due to AIDS, CVD or other causes (Figure S1).

As for the stratification of CVD risk, we referred to the 10-year ASCVD risk assessment scheme from the Chinese Guidelines for Lipid Management (2023) [31]. This assessment, which uses low-density lipoprotein cholesterol (LDL-C) and total cholesterol (TC) as core serum biomarkers alongside traditional risk factors, classifies individuals as low risk (< 5% for CVD incidence), intermediate risk (5%–9% for CVD incidence), or high risk (≥ 10% for CVD incidence) for a MACE within 10 years. Transitions between health states were determined based on CVD risk levels and disease progression. In each cycle, individuals who have never suffered from CVD could experience a first-onset CVD event, with incidence rates strictly aligned with the risk tiers defined in the guidelines. After the first CVD event (MACE), individuals transitioned to a “post-first CVD” state, characterized by an elevated risk of recurrence and increased CVD-related mortality. Those experiencing recurrent CVD events moved to a “post-recurrent CVD” state, reflecting further disease burden and persistent mortality risk. The model also accounted for dynamic transitions between risk tiers, allowing movement across CVD risk levels over time (Figure S1). Specifically, risk tier reclassification occurred at scheduled screening rounds—every 6, 12, or 24 months depending on the strategy—using updated age and lipid profiles via the China-PAR risk equation (Supplementary Materials, Sect.  3.2).

### Strategies for CVD primary prevention

We developed a hierarchical CVD primary prevention model for PWH in China. Pitavastatin 4 mg/day was served as the foundational preventive drug for all eligible individuals, with intervention intensification being proportional to CVD risk levels [32]. These regimens have demonstrated high efficacy in CVD prevention and are recommended by Chinese guidelines [33]. We defined the current screening frequency of CVD risk and statins uptake as the status quo: only about 11% of individuals at substantial (medium/high) CVD risk has screened for CVD and used statins for primary prevention each year, with no treatment intensification implemented in practice [34]. This rate was applied uniformly across both risk strata. For the status quo, we assumed a 24-month screening interval and a baseline statin adherence of 50%, consistent with the strategy definitions and published estimates for primary prevention populations [24].

To explore the optimal strategies in the context of limited resource, we simulated a cohort of 100,000 PWH to compare a series of interventions for CVD prevention. Each strategy (except for the status quo) was characterized by three main scenarios: (i) screening intervals for routine CVD risk assessments (every 6, 12, or 24 months); (ii) the lipid-lowering intensification regimen for individuals identified as high-risk (pitavastatin add-on ezetimibe (abbreviated as PE), pitavastatin add-on PCSK9 inhibitor (abbreviated as PC), or continuation of pitavastatin monotherapy (abbreviated as PP)), while pitavastatin monotherapy was adopted for all individuals at medium CVD risk by default; (iii) whether or not an electronic pillbox was deployed to enhance medication adherence among medium- and high-risk individuals (use the prefix “E” to indicate those who have used electronic pillboxes). For example, strategy “E-PE-6” represents 6-monthly screening, pitavastatin alone for medium-risk, pitavastatin+ezetimibe for high-risk, and applying electronic pillbox for adherence enhancement (See Table S1 for details of all strategies and their corresponding labels). As a conservative approach, any potential improvements in screening technology, background adherence rates (unless the pillbox was part of the strategy), and reductions in drug costs were not considered over the modelling period.

### Data source

Model parameters were derived from published literature and public data on CVD epidemiology, with a focus on the Chinese population (Table S2). Health-related parameters included CVD incidence and mortality rates, disease state transition probabilities, and utility values, all obtained from published literature to simulate the natural history of CVD and intervention effects in PWH. Background mortality data were sourced from the China Statistical Yearbook 2024 [35]. The CVD risk stratification criteria for the initial cohort, baseline lipid levels across different risk categories, and demographic characteristics were all obtained from the China Cardiovascular Disease High-Risk Population Early Screening and Comprehensive Intervention Study [34].

We estimated CVD incidence rates among PWH across different risk strata based on data from the international multicenter REPRIEVE trial, which included Chinese participants [12]. The study enrolled PWH receiving ART with low to high CVD risk, evaluating the preventive effect of pitavastatin through a randomized controlled design, with MACE as the primary endpoint, including cardiovascular death, myocardial infarction, and revascularization. Based on the trial, we determined the annual incidence rates across different risk levels: 0.44 per 100 PYs for low-risk group, 1.15 per 100 person-years (PYs) for medium-risk group, and 1.75 per 100 PYs for high-risk group. Additionally, according to a systematic review that incorporated data from Chinese studies that focused on clinical outcomes after acute coronary syndrome or revascularization in PWH, the CVD recurrence rate was determined to be 13.8 per 100 PYs [36]. All annual event rates were converted to monthly probabilities for the monthly-cycle model (See Supplementary materials).

As the effectiveness of statins was highly dependent on adherence, we incorporated parameters of adherence for that in baseline and with electronic pillbox support. Baseline statin adherence, derived from a systematic review of large-scale pharmacy records with ≥ 1-year follow-up, was set at 50% (41%–69%) without intervention [24]. For the electronic pillbox group, adherence was set at 80% (74%–83%) based on a randomized controlled trial in tuberculosis patients [26]. As this effect may not directly generalize to PWH on statins, we varied adherence over a wide range (60%–90%) in sensitivity analyses. Regarding intervention efficacy, we established LDL-C reduction percentages for each regimens: pitavastatin (4 mg monthly) reduced LDL-C by 10.37% (4.1%-15.7%) [37], the combination of statin-ezetimibe by 23.60% (21.7%-25.6%) [38], and the combination of statin-PCSK9 inhibitor by 57.93% (54.91%-60.95%) [39]. Each 1 mmol/L reduction in LDL-C was associated with a 21.0% (19.0%-23.0%) reduction in CVD risk [40]. In actual effectiveness calculations, we adjusted theoretical efficacy using adherence parameters. Specifically, the actual CVD risk reduction for each intervention regimen was calculated by first determining the theoretical risk reduction based on its LDL-C reduction percentage through risk conversion coefficients, then applying weighting adjustments according to actual adherence levels.

Cost data were collected from the perspective of the healthcare system, consisting of the direct medical costs of statin medications, CVD risk assessment and related testing, electronic pillbox, treatment for MACE and HIV, and adverse event management (Table S2). For medication therapy, we considered three specific regimens: statin monotherapy, statin-ezetimibe combination therapy, and statin–PCSK9 inhibitor combination therapy. The associated medication costs were derived from a published Chinese cost-effectiveness analysis [41]. For the electronic pillbox, the procurement and maintenance costs were referenced against the Chinese digital health device market. The device cost was set at a one-time investment of $51.67 per unit, with an assumed lifespan of 2–5 years, after which replacement would be required. A monthly maintenance fee of $23.42 was applied for ongoing operational support, including platform access, technical support, and clinical monitoring [42]. Regarding adverse event management, the annual treatment cost for statin-associated diabetes was $1,139.75, and the annual treatment cost for myopathy was $1,825.42 [41]. All cost data were derived from China’s healthcare service pricing system, centralized drug procurement prices, and the China Health Statistics Yearbook, and were uniformly converted to 2025 US$. All future costs were discounted at 3% annually to present values in our model.

### Cost-effectiveness analysis

We evaluated the effectiveness and cost-effectiveness of various CVD primary prevention strategies based on multiple model outputs. Health effects were measured as quality-adjusted life years (QALYs), calculated by combining the utilities of specific health states with their corresponding duration in years. All future QALYs were discounted at 3% annually to present values in our model. Other health outcomes assessed included the number of first MACE averted, MACE-free years of each strategy and CVD-related deaths.

The incremental cost-effectiveness ratio (ICER) between different strategies, measured as cost per QALY gained, was calculated to compare the cost-effectiveness between the status quo and alternative screening approaches under certain willingness-to-pay (WTP) threshold. The WTP threshold was set at 1 x GDP per capita ($13,445) and 3 × GDP per capita for China ($40,335) based on WHO-CHOICE recommendations and previous publications [43], to examine how changes in WTP thresholds might affect the cost-effectiveness conclusions. Net monetary benefit (NMB, calculated as incremental effectiveness × willingness-to-pay threshold, minus incremental cost) was applied to measure the optimal strategy under different willingness-to-pay thresholds. The strategy with the highest NMB value was defined as the preferred/optimal strategy at the specified WTP threshold. The benefit-cost ratio (BCR), defined as the return in dollars on medical cost savings per dollar invested in CVD screening and risk management, was also applied to evaluate the budget impact of the implemented strategies. BCR was calculated as the number of dollars saved in direct medical costs of CVD treatment for every dollar invested in CVD prevention: BCR ≥ 1 indicates that intervention has net economic benefits, while BCR < 1 implies that benefits are insufficient to cover the costs.

We also conducted extensive sensitivity analysis to determine the effects of input parameter uncertainties. One-way sensitivity analysis presented as tornado diagrams assessed the impact of changes in a single parameter on the cost-effectiveness of the optimal strategy. Probabilistic sensitivity analysis (PSA) with 1,000 Monte Carlo simulations was applied to evaluate the combined uncertainty of all model parameters on ICERs across a wide range of WTP thresholds. Our study adheres to the reporting guidelines outlined in the Consolidated Health Economic Evaluation Reporting Standards (CHEERS 2022) statement (see Supplementary Material: CHEERS Checklist) [44].

## Results

### Baseline results

**Fig. 1:**
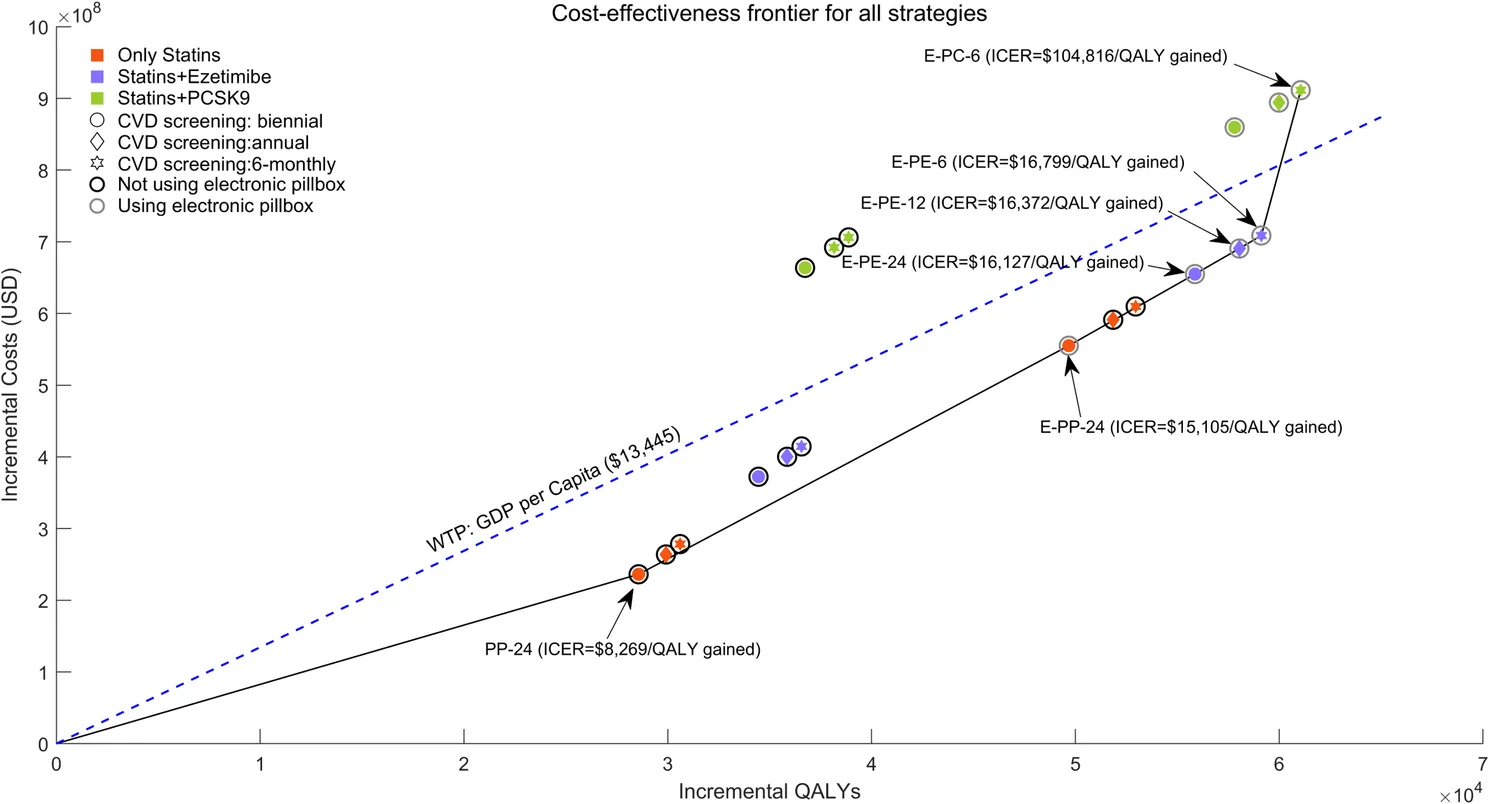
Cost-effectiveness planes for all strategies among PWH in China. This figure presents a standard cost-effectiveness plane to visualize the efficiency of different strategies relative to the status quo. Each marker represents a unique prevention strategy. The X-axis shows the incremental quality-adjusted life years (QALY) gained compared to status quo, and the Y-axis shows the incremental cost (US$) compared to status quo. The solid black line connecting several strategies forms the efficiency frontier, representing the set of non dominated strategies. The incremental cost effectiveness ratio (ICER) values shown are calculated versus the prior, more cost-effective strategy on the frontier. The dashed diagonal line indicates the willingness to pay (WTP) threshold of $13,445 (1× GDP per capita) used in this analysis. All costs reported in US$, unless otherwise stated. For each scenario, CVD prevention strategy was either pitavastatin add-on ezetimibe (abbreviated as PE), pitavastatin add-on PCSK9 inhibitor (abbreviated as PC), or continuation of pitavastatin monotherapy (abbreviated as PP)); CVD risk screening frequency was either 6-monthly (6), 12-monthly (12), or 24-monthly (24); using electronic pillbox (E) or not. GDP=gross domestic product. CVD=cardiovascular disease

If current practice (status quo) was maintained for CVD primary prevention among PWH, a simulated cohort of 100,000 individuals would experience 40,195 first MACE episodes at a total cost of $3297.5 million over the modelling time horizon. The E-PC-6 strategy (6-monthly screening, pitavastatin alone for medium-risk individuals, pitavastatin+PCSK9i for high-risk individuals, with electronic pillbox use) resulted in the highest total cost of $3470.9 million, an increase of $173.4 million (5.3%) over the status quo (Table 1). The additional cost compared to the status quo was primarily driven by the extensive use of PCSK9i (accounting for approximately $1199.9 million in drug costs) and the implementation of electronic pillboxes. E-PC-6 also yielded the greatest health benefits, gaining 60,159 QALYs and averting 10,571 first MACE episodes, corresponding to a 26.3% relative reduction in MACE incidence. However, due to high cost in medication, this strategy was not cost-effective with its ICER value far beyond the WTP threshold in China ($104,816/QALY gained) (Fig. 1).

**Table 1:** Base-case results of cost-effectiveness analysis for all strategies (a cohort of 100,000 PWH)

Strategy^&^	Cost, $, million			Effectiveness			Cost-effectiveness				
	CVD screening	Statins use	Treatment	No. first MACE	MACE-free years (per 100 PYs)	QALYs	Cost per first MACE case averted, $^a^	ICER ($/QALY gained)^b^	BCR^c^	NMB1^d^	NMB2^e^
Status quo^*^	632.79	1.66	2775.26	40,195	69.91	1,575,034	…	…	…	177,666	601,193
PP-24	826.16	124.13	2695.66	35,897	72.54	1,603,604	54,949	8,269	1.36	179,145	610,354
PP-12	853.02	126.34	2694.19	35,779	72.64	1,604,945	59,751	20,570	1.25	179,049	610,619
PP-6	866.69	127.34	2694.05	35,722	72.69	1,605,640	62,237	20,919	1.20	178,997	610,754
PE-24	832.71	366.89	2582.37	34,611	73.08	1,609,486	66,874	24,413	1.12	178,576	611,366
PE-12	859.62	368.98	2581.24	34,492	73.18	1,610,890	70,164	19,850	1.07	178,486	611,654
PE-6	873.29	369.71	2581.29	34,436	73.23	1,611,598	71,988	20,408	1.04	178,436	611,795
E-PP-24	849.5	645.29	2469.99	31,592	74.46	1,624,710	64,521	15,105	1.16	178,795	615,679
E-PP-12	877.06	655.43	2468.42	31,408	74.61	1,626,890	67,286	16,582	1.11	178,726	616,197
E-PP-6	891.05	659.92	2468.50	31,322	74.69	1,627,992	68,730	16,828	1.09	178,689	616,456
E-PE-24	856.18	819.43	2389.04	30,285	75.01	1,630,903	66,093	16,127	1.13	178,628	617,178
PC-24	835.23	902.66	2335.50	34,119	73.29	1,611,770	109,216	Dominated	0.68	175,968	609,373
E-PE-12	883.71	828.67	2387.90	30,107	75.17	1,633,080	68,452	16,372	1.09	178,565	617,700
PC-12	862.15	904.40	2335.06	33,999	73.39	1,613,195	111,669	Dominated	0.67	175,878	609,666
PC-6	875.82	904.52	2335.45	33,944	73.44	1,613,906	112,949	Dominated	0.66	175,832	609,811
E-PE-6	897.67	832.62	2388.13	30,026	75.24	1,634,159	69,687	16,799	1.07	178,529	617,954
E-PC-24	858.26	1191.42	2219.60	29,880	75.19	1,632,848	83,318	Dominated	0.90	176,844	615,916
E-PC-12	885.78	1198.79	2219.23	29,704	75.34	1,635,021	85,222	215,059	0.88	176,791	616,448
E-PC-6	899.73	1201.59	2219.77	29,624	75.41	1,636,093	86,235	104,816	0.87	176,762	616,707

&: For each scenario, CVD prevention strategy was either pitavastatin add-on ezetimibe (abbreviated as PE), pitavastatin add-on PCSK9 inhibitor (abbreviated as PC), or continuation of pitavastatin monotherapy (abbreviated as PP)); CVD risk screening frequency was either 6-monthly (6), 12-monthly (12), or 24-monthly (24); using electronic pillbox (E) or not. *Status quo: only 11% of individuals at medium/high CVD risk would screen for CVD and use statins for primary prevention. a: Incremental cost per first MACE averted vs. status quo; b: ICERs are calculated by comparing each strategy to the next less costly, non-dominated strategy; c: BCR (benefit to cost ratio). In a certain statin strategy, BCR=number of dollars saved from MACE treatment/number of dollars invested in statin using; d: NMB1 (net monetary benefit) at a willingness-to-pay threshold of 1× GDP per capita = effectiveness × (1× GDP per capita) – cost; e: NMB2 at a WTP threshold of 3× GDP per capita = effectiveness × (3× GDP per capita) − cost. NMB values are reported in thousands of US dollars (×$1,000) at the cohort level over the lifetime horizon. QALY: quality adjusted life years; ICER: incremental cost-effectiveness ratio. PYs: Person-years

### Cost-effectiveness metrics

When NMB at the stated WTP threshold was used as the primary decision criterion, the optimal strategy changed with the societal value placed on health. At a WTP threshold of 1× GDP per capita ($13,445), the optimal strategy was PP-24 (24-monthly screening, pitavastatin alone for both medium-risk and high-risk groups, no pillbox use). This strategy gained 28,570 QALYs, averted 4,298 first MACE episodes (a 10.7% reduction compared with the status quo), and had an ICER of $8,269 per QALY gained. At the higher WTP threshold of 3× GDP per capita ($40,335), E-PE-6 became the most cost-effective strategy (ICER: $16,799/QALY gained). This superior effectiveness stemmed from the optimal balance of a cost and effectiveness of potent lipid-lowering regimen and significant adherence enhancement, even though the most expensive treatment (PCSK9i) was not used (Fig. 1).

Other metrics provide complementary insights for different stakeholders. The BCR, which reflects the return on investment from averted MACE treatment costs, ranged from 0.87 (E‑PC‑6) to 1.36 (PP‑24). Strategies that incorporated electronic pillboxes or PCSK9 inhibitors—such as E‑PC‑6 (BCR = 0.87) and PC‑24 (BCR = 0.68)—consistently yielded BCR values below 1. In contrast, non‑digital standard statin‑based strategies, notably PP‑24 (BCR = 1.36) and PP‑12 (BCR = 1.25), produced BCRs above 1. The ratio varied inversely with the intensity and technological augmentation of the intervention.

### Results of sensitivity analysis

**Fig. 2:**
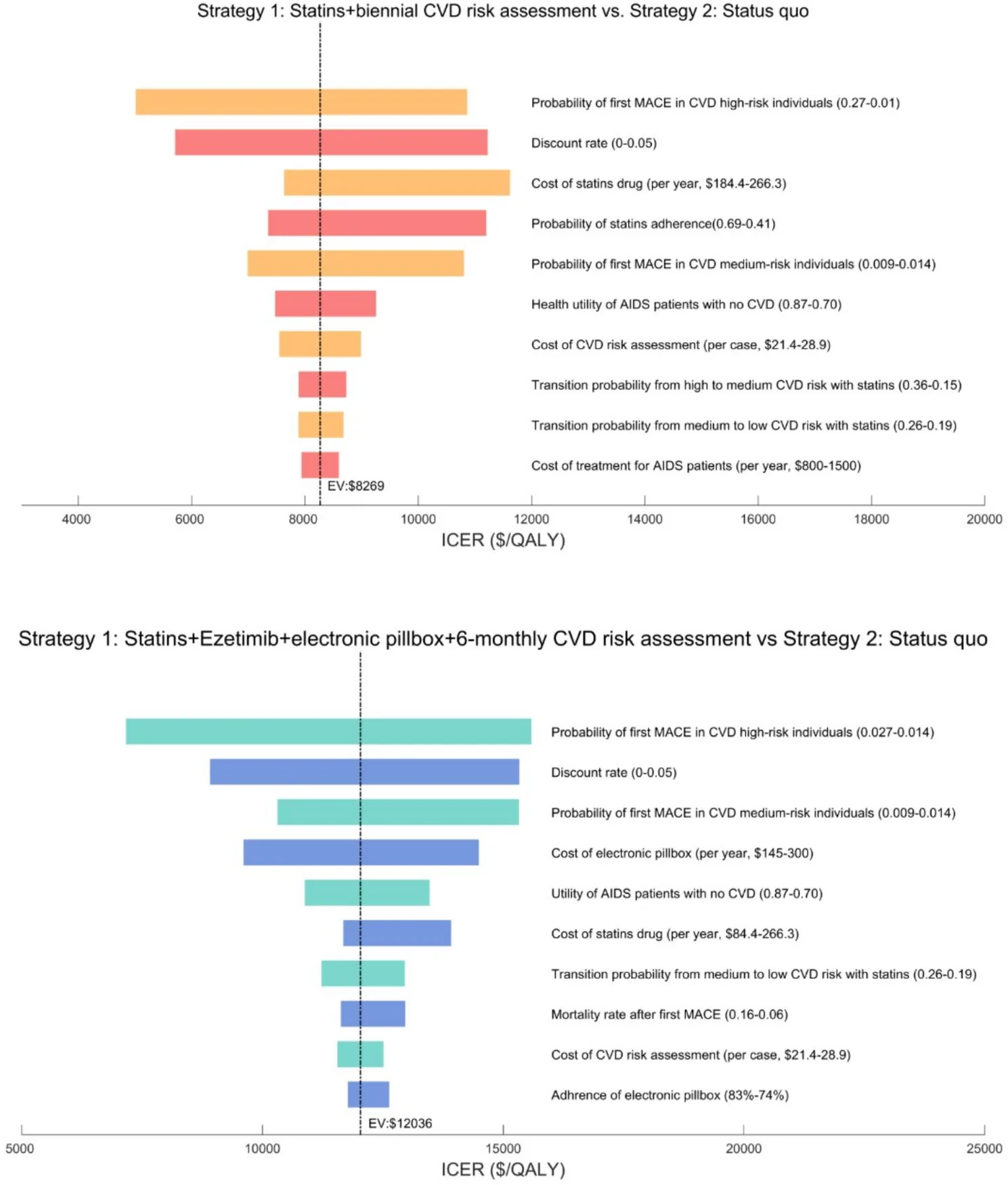
One-way sensitivity analyses of optimal strategies among PWH in China

To verify the robustness of our findings, we performed extensive sensitivity analyses, which confirmed that the baseline results remained stable across a wide range of parameter values (Fig. 2). One-way sensitivity analysis identified key drivers of cost-effectiveness when comparing the optimal strategy to the status quo at different WTP thresholds. At the lower threshold of 1 × GDP per capita ($13,445), the ICER of the PP-24 strategy was most sensitive to the probability of first MACE in CVD high-risk individuals and medication adherence rates in the medium- and high-risk groups. At the higher threshold of 3 × GDP per capita ($40,335), the ICER of the E-PE-6 strategy was most strongly influenced by medication adherence rates and the unit cost of ezetimibe. Variations in these parameters within their plausible ranges could alter the cost-effectiveness conclusion, underscoring their importance in real-world implementation.

**Fig. 3:**
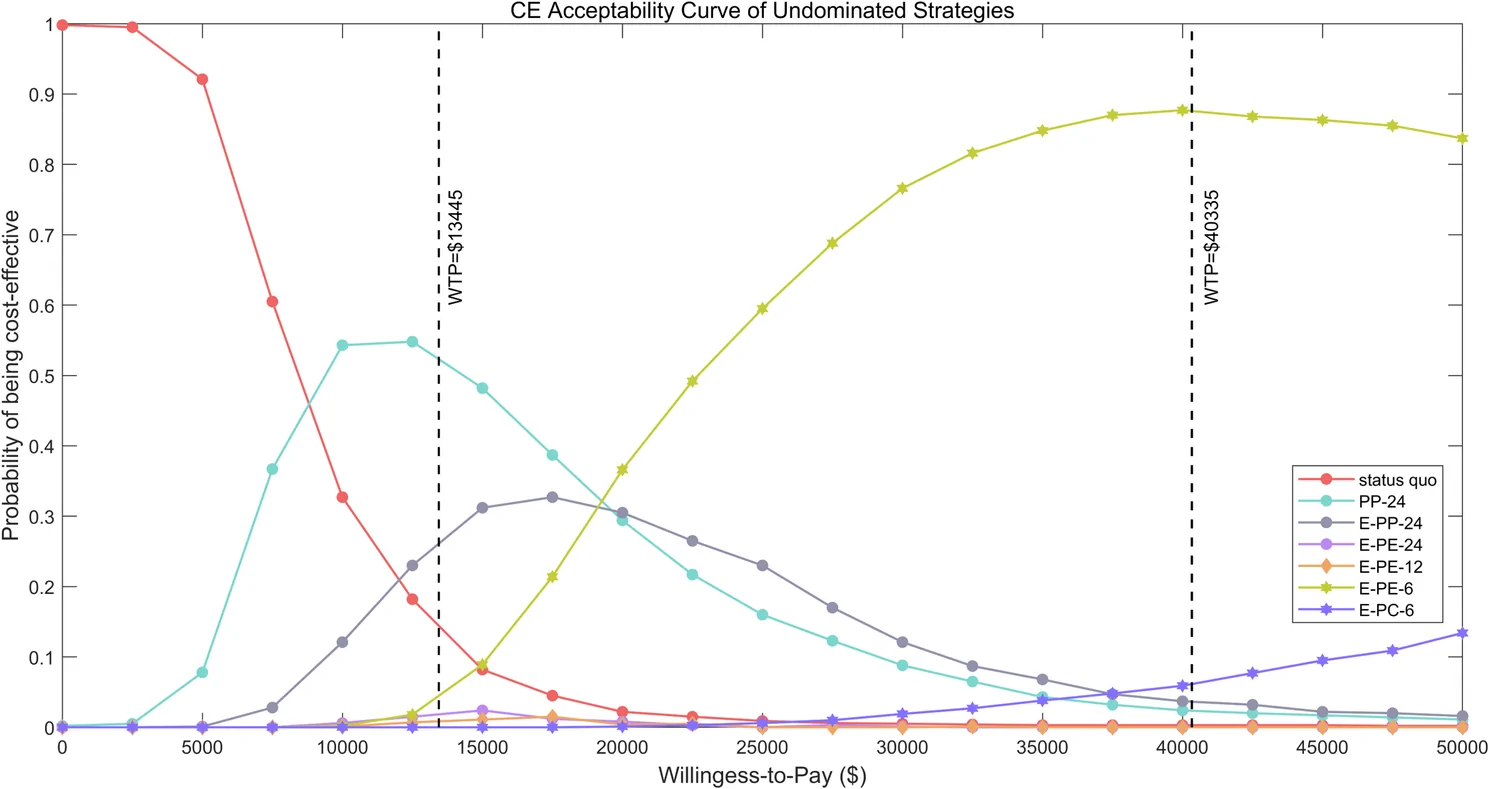
Cost-effectiveness acceptability curves for all undominated strategies among PWH in China

Probabilistic sensitivity analysis based on 1,000 Monte Carlo simulations further validated the robustness of the primary findings. The ICER for PP-24 versus status quo was $8,269/QALY (95% UI: $6,543–$10,124), and for E-PE-6 versus status quo was $16,799/QALY (95% UI: $13,872–$20,015). At the WTP threshold of 1 × GDP per capita, the PP-24 strategy remained the highest probability of being cost-effective (52.1%), although the remaining 47.9% probability was distributed across multiple alternative strategies (e.g., PP-12 and E-PP-24), indicating substantial decision uncertainty at this threshold. At the higher threshold of 3 × GDP per capita, the E-PE-6 strategy still demonstrated the highest probability of being cost-effective (85.6%). These results confirmed that the optimal strategies identified in the base-case analysis are robust under parameter uncertainty across the specified WTP thresholds (Fig. 3).

## Discussion

Our cost-effectiveness analysis evaluated enhanced strategies for CVD prevention against the current standard of care in China. Using a NMB framework at standard WTP thresholds, we found the optimal strategy depended on the societal value placed on a unit of health gain. At a WTP threshold of 1× GDP per capita, the optimal approach was to implement 24-monthly screening with tiered pharmacotherapy (pitavastatin for both medium-risk and high-risk groups) but without electronic pillbox (PP-24). In contrast, at the higher WTP threshold of 3× GDP per capita, the optimal strategy shifted to a more intensive regimen of pitavastatin in combination with ezetimibe, along with 6-monthly CVD risk screening, and adding an electronic pillbox to support medication adherence.

China currently lacks dedicated national guidelines for cardiovascular prevention in PWH. Current cardiovascular prevention guidelines commonly recommend annual risk assessments for high-risk groups, yet the optimal screening frequency for PWH remains undefined, with practice often relying on this general advice [45]. To address this gap, our modeling analysis demonstrates a dynamic relationship between intervention intensity and monitoring frequency. We found that more intensive regimens, such as statins combined with ezetimibe therapy, produce greater cardiovascular risk reduction. This allows for safely extending screening intervals—for example, from every 6 to 24 months—while maintaining health outcomes, a principle we term “intervention-for-monitoring efficiency”. The mechanistic rationale is that greater LDL-C reduction lowers residual CVD risk to a greater extent, meaning that age-related risk progression takes longer to reach the threshold at which treatment intensification becomes warranted. As a result, extending screening intervals does not compromise timely detection of clinically meaningful risk escalation. This relationship enables stratified management: high-risk individuals on intensive therapy may require less frequent monitoring, while those on standard therapy benefit from closer follow-up. Our cost-effectiveness analysis substantiates this logic. Under constrained resources or lower societal WTP, the most efficient approach involves low-frequency screening paired with standard therapy, as in the PP-24 strategy. When greater investment is feasible, transitioning to high-frequency screening alongside intensive therapy, as in the E-PE-6 strategy, yields superior health gains. This evidence-based, tiered framework aligns with risk-stratified prevention principles and offers a pragmatic model for personalized care in PWH. This framework accommodates China’s decentralized HIV care system, enabling provinces to select strategies according to local resource availability; it requires integration of electronic pillboxes into existing HIV case management platforms; and scaling up to the estimated 1.2 million statin-eligible PWH in China would necessitate dedicated financing mechanisms, such as inclusion in the National Reimbursement Drug List. Our ICERs are substantially lower than those reported in high-income settings (e.g., $35,000–$55,000/QALY in US PWH), reflecting China’s lower drug and labor costs [29].

When assessing the cost-effectiveness of various screening and intervention strategies, our model innovatively incorporates real-world medication adherence as a central variable. This is particularly critical for PWH, who often manage complex long-term medication regimens. Sensitivity analysis reveals that adherence rate is among the most influential parameters determining the economic viability of all prevention strategies. Our findings implied that to realize the projected health gains of optimized approaches such as E-PE-6, investments in improving medication adherence may be equally as vital as the selection of the pharmacological regimen itself. To address this adherence challenge, digital health tools—such as electronic pillboxes—provide a practical and evidence-based solution. Studies demonstrate that interventions featuring medication reminders, behavioural feedback, and data connectivity can significantly improve long-term adherence in chronic disease management [26, 46]. Notably, while our model conservatively focused on its application for statin adherence, the same electronic pillbox could also be used to manage adherence to ART regimen in HIV management. Although this synergistic effect for integrated comorbidity management was not analysed, it highlights the tool’s potential for broader real-world impact among PWH with chronic conditions. This is especially relevant as our model identifies ezetimibe-based intensive therapy as the most cost-effective pharmacologic option, yet its success is entirely contingent upon sustained patient commitment to daily oral dosing. Consequently, we proposed a tiered implementation framework: in settings with higher willingness-to-pay, a strategy of “ezetimibe-based intensive therapy combined with digital adherence support” (consistent with the E-PE-6 logic) is recommended for high-risk individuals. In resource-constrained environments, a strategy of “standard therapy with low-frequency monitoring and basic adherence support” (following the PP-24 logic) represents a more feasible alternative. Ultimately, translating the health benefits demonstrated in our model into real-world outcomes depends on building an integrated management system that systematically links risk screening, intervention selection, adherence support, and long-term follow-up.

Despite the strengths, our study has several limitations. First, as China has not established its own WTP threshold for health interventions, we adopted the conventional benchmark of 3 times the GDP per capita in our base-case analysis—a commonly applied yet debated reference in health economic evaluations. To enhance the robustness and policy relevance of our findings, we also conducted sensitivity analyses using a recently proposed, empirically derived threshold of 1 time GDP per capita for China, and presented cost-effectiveness acceptability curves across a broad spectrum of WTP values. This approach allows decision-makers to assess the probability of each strategy being cost-effective under different resource constraints, though future work is urgently needed to establish an official, context-specific WTP threshold for China. Second, our model did not incorporate the potential impact of improved cardiovascular risk management on secondary outcomes, such as quality-of-life adjustments for non-cardiovascular conditions or indirect economic benefits from productivity gains. While this simplification offers a more conservative and focused estimate of the direct cost-effectiveness of the screening and intervention strategies evaluated, it may lead to an underestimation of their full societal value.

Third, the key parameters in our model regarding the effectiveness of digital health interventions (such as electronic pillboxes) in improving medication adherence were primarily derived from studies conducted in general chronic disease populations or non-HIV-specific cohorts. Currently, high-quality empirical data on the long-term adherence improvement and subsequent impact on cardiovascular outcomes specifically for PWH using such tools remain limited. We addressed this uncertainty by varying the magnitude of adherence improvement over a wide range in our sensitivity analyses. However, future targeted intervention studies within the PWH population are needed to obtain more accurate parameter estimates. Fourth, our analysis focused on evaluating discrete, defined screening and intervention strategies (such as E-PE-6 and PP-24) rather than simulating a fully continuous or dynamic mix of approaches that might be used in practice. While this provides clear contrast between structured policy options, real-world application would likely involve more flexible, individualized adaptation. Fourth, because our model was based on current epidemiological and clinical data, it did not account for future changes in HIV care, cardiovascular treatment paradigms, or the potential introduction of novel prevention technologies. As such, our findings represent an assessment under contemporary conditions and may serve as a conservative baseline for evaluating future innovations. Fifth, we did not include training or programmatic overhead costs beyond direct device and support expenses, which may slightly underestimate implementation costs but is unlikely to alter the relative strategy ranking.

In conclusion, our model supports a risk-stratified approach to CVD prevention among PWH in China, integrating periodic screening with tailored lipid-lowering therapy. The preferred strategy was threshold-dependent: PP-24 (without pillboxes) is preferred at 1× GDP per capita, while E-PE-6 (with pillboxes) becomes cost-effective at 3× GDP per capita. Incorporating electronic pillboxes into HIV care offers a cost-effective pathway to translate statins trial efficacy into population-level health impact in settings with greater healthcare investment. These findings also advocate for urgent integration of tiered CVD prevention into HIV care platforms, particularly within LMICs undergoing epidemiologic transition. However, realizing these modelled benefits demands implementation research to generate HIV-specific adherence data and evaluate digital interventions within integrated chronic care models, ultimately establishing medication adherence as a modifiable implementation outcome as pivotal as pharmacological selection itself for non-communicable disease control in aging HIV populations globally.

## Supplementary Information

Below is the link to the electronic supplementary material.

Supplementary Material 1

## Data Availability

The datasets used and/or analysed during the current study are available from the corresponding author on reasonable request.

## Material and/or code availability

The Markov model was provided to the manuscript reviewers during the peer review process; however, the model and any associated code are proprietary and will not be available in the public domain.
