# Supplementary material for: Cost-effectiveness of statin-based primary prevention of cardiovascular disease with electronic pillboxes for people with HIV in China
Source: BMC Health Serv Res. 2026 Aug 4;26:1514. doi: 10.1186/s12913-026-15173-y (PMC13732939; doi:10.1186/s12913-026-15173-y)
Supplement: Supplementary file 1 [file 12913_2026_15173_MOESM1_ESM.docx]

**Supplementary Materials**

This supplementary document describes in detail the screening strategies, model structure, parameter estimation, and uncertainty and sensitivity analyses.

Contents

[1. Model Structure	2](#_Toc220522510)

[2. Screening Strategies	4](#_Toc220522511)

[3. Model Parameter Estimation	5](#_Toc220522512)

[3.1 Core Clinical and Economic Parameters	5](#_Toc220522513)

[3.2 Dynamic Risk Factor Progression and Risk Calculation	7](#_Toc220522514)

# 1. Model Structure

We developed a deterministic static Markov model to simulate the progression of cardiovascular disease (CVD) among people with HIV (PWH) in China. The model aimed to evaluate the cost-effectiveness of a digital technology-driven hierarchical screening strategy for CVD prevention in this population. We simulated a static cohort of 100,000 individuals using monthly cycles from model initiation until age 85. The model incorporated three key dimensions: a) HIV disease status, b) CVD risk stratification (categorized as low, medium, or high risk according to Chinese guidelines), and c) CVD clinical status.

Transitions between health states were determined by CVD risk levels and disease progression. Individuals without CVD history could experience first-onset events at rates corresponding to their risk stratum. After an initial event, patients moved to a "post-first CVD" state with elevated recurrence risk and CVD-related mortality. Subsequent recurrent events transitioned individuals to a "post-recurrent CVD" state with further increased disease burden. The model also incorporated dynamic transitions between risk strata over time.

Each state in our model could transition to another state based on specific transition probabilities. Rectangles represent Markov health states, and lines with arrowheads represent transitions and their directions. Notably, arrows with arcs indicate that individuals would remain in their current states during the next cycle according to specific probabilities. The model treated death as an absorbing state and continued until all individuals either reached age 85 or entered this terminal state. This framework enabled the evaluation of long-term outcomes of different screening and intervention strategies for cardiovascular health in people with HIV (Figure S1).

**Figure S1 The Markov model for cardiovascular disease progression among PWH**

Risk tier transitions (between low, intermediate, and high risk) occur at scheduled screening rounds (every 6, 12, or 24 months) based on updated age and lipid profiles; transitions to CVD events and death can occur in any monthly cycle.

# 2. Screening Strategies

To simulate the diverse combinations of CVD screening frequencies and treatment adherence interventions, we incorporated the following key elements in our modeling strategies: (a) three screening frequencies, specifically standardized CVD risk assessments every 6, 12, or 24 months; (b) adherence monitoring interventions targeting medium- and high-risk groups, primarily through electronic medication monitors (electronic pillbox); and (c) treatment intensification regimens based on risk stratification, including add-on ezetimibe or PCSK9 inhibitors for high-risk individuals. All strategies were compared against the current standard of care, characterized by a uniform 24-month screening interval without systematic adherence monitoring.

Specifically, screening strategies were differentiated according to individuals' CVD risk levels. For medium- and high-risk groups, various combinations of screening intervals and electronic pillbox interventions were tested to evaluate their cost-effectiveness. Beyond foundational statin therapy, treatment intensification with combination regimens was further evaluated for high-risk populations. This stratified approach aimed to identify the most resource-efficient combinations of screening and interventions for specific risk subgroups (Table S1). Detailed parameters and descriptions of all strategies implemented among people with HIV in China are provided in Supplementary Table S2.

**Table S1 Comprehensive Overview of Simulated CVD Screening and Treatment Strategies**

| **Strategy^#^** | **Screening Frequency (months)** | **Using electronic pillboxes^a^** | **For medium-risk individuals** | **For high-risk individuals** |
| --- | --- | --- | --- | --- |
| PP-6 | 6 | No | Pitavastatin 4mg QD | Pitavastatin 4mg QD |
| PE-6 | 6 | No | Pitavastatin 4mg QD | Pitavastatin 4mg QD + Ezetimibe 10mg |
| PC-6 | 6 | No | Pitavastatin 4mg QD | Pitavastatin 4mg QD + PCSK9 inhibitor |
| E-PP-6 | 6 | Yes | Pitavastatin 4mg QD | Pitavastatin 4mg QD |
| E-PE-6 | 6 | Yes | Pitavastatin 4mg QD | Pitavastatin 4mg QD + Ezetimibe 10mg |
| E-PC-6 | 6 | Yes | Pitavastatin 4mg QD | Pitavastatin 4mg QD + PCSK9 inhibitor |
| PP-12 | 12 | No | Pitavastatin 4mg QD | Pitavastatin 4mg QD |
| PE-12 | 12 | No | Pitavastatin 4mg QD | Pitavastatin 4mg QD + Ezetimibe 10mg |
| PC-12 | 12 | No | Pitavastatin 4mg QD | Pitavastatin 4mg QD + PCSK9 inhibitor |
| E-PP-12 | 12 | Yes | Pitavastatin 4mg QD | Pitavastatin 4mg QD |
| E-PE-12 | 12 | Yes | Pitavastatin 4mg QD | Pitavastatin 4mg QD + Ezetimibe 10mg |
| E-PC-12 | 12 | Yes | Pitavastatin 4mg QD | Pitavastatin 4mg QD + PCSK9 inhibitor |
| PP-24 | 24 | No | Pitavastatin 4mg QD | Pitavastatin 4mg QD |
| PE-24 | 24 | No | Pitavastatin 4mg QD | Pitavastatin 4mg QD + Ezetimibe 10mg |
| PC-24 | 24 | No | Pitavastatin 4mg QD | Pitavastatin 4mg QD + PCSK9 inhibitor |
| E-PP-24 | 24 | Yes | Pitavastatin 4mg QD | Pitavastatin 4mg QD |
| E-PE-24 | 24 | Yes | Pitavastatin 4mg QD | Pitavastatin 4mg QD + Ezetimibe 10mg |
| E-PC-24 | 24 | Yes | Pitavastatin 4mg QD | Pitavastatin 4mg QD + PCSK9 inhibitor |

^a^Using electronic pillbox: when applied, is targeted at medium- and high-risk groups to monitor and improve medication adherence. ^#^Strategy codes: The first **E** represents whether using electronic pillboxes. The second letter represents regimen for medium-risk individuals (P = Pitavastatin monotherapy). The third letter specifies the intensified pharmacologic regimen for high-risk individuals (P = Pitavastatin monotherapy, E = Pitavastatin + Ezetimibe, C = Pitavastatin + PCSK9 inhibitor. The trailing number denotes the screening interval in months (e.g., -12 = 12-month screening). Example: E-PE-12 = 12-month CVD risk screening, pitavastatin alone for medium-risk individuals, and pitavastatin+ezetimibe for high-risk individuals, with electronic pillbox use for adherence enhancement.

# 3. Model Parameter Estimation

The model parameters are derived from two main components: (a) the core clinical and economic parameters that govern the Markov state transitions, costs, and health utilities; and (b) the dynamic risk factor progression parameters that enable the annual updating of individual CVD risk.

## 3.1 Core Clinical and Economic Parameters

This set of parameters forms the foundation of the Markov model's structure and economic evaluation. It includes the baseline distribution of the PWH cohort across CVD risk strata and their corresponding lipid profiles, the incidence rates of first and recurrent CVD events stratified by risk level, the efficacy and adherence rates associated with different lipid-lowering regimens (e.g., pitavastatin monotherapy, and combinations with ezetimibe or PCSK9 inhibitors), state-specific utility weights, and all relevant costs (e.g., medications, risk assessments, and CVD event management). A full listing of these parameters, their baseline values, uncertainty ranges, probability distributions for probabilistic sensitivity analysis (PSA), and sources is provided in Table S2.

**Table S2 Core model parameters, baseline values, and sources.^a^**

| **Parameter** | **Base Case** | **Range** | **Distribution for PSA** | **Ref.** |
| --- | --- | --- | --- | --- |
| *Cohort Characteristics* |  |  |  |  |
| CVD Risk Stratum (%) |  |  |  | [1] |
| Low risk (<5%) | 54.30 | ... | ... |  |
| Medium risk (5-10%) | 38.50 | ... | ... |  |
| High risk (≥10%) | 7.20 | ... | ... |  |
| Lipid Profile, Mean (SD), mmol/L |  |  |  | [2] |
| LDL-C, Low risk | 2.20 (0.78) | ... | ... |  |
| LDL-C, Medium risk | 3.00 (0.77) | ... | ... |  |
| LDL-C, High risk | 4.15 (1.09) | ... | ... |  |
| HIV/AIDS Status (%) |  |  |  | [3] |
| HIV patients | 63.93 | ... | ... |  |
| AIDS patients | 36.07 | ... | ... |  |
|  |  |  |  |  |
| *Intervention Efficacy & Adherence* |  |  |  |  |
| LDL-C Reduction (%) |  |  |  |  |
| Pitavastatin 4mg | 10.37 | 4.10-15.70 | Beta(23.98, 207.56) | [4] |
| Ezetimibe + Statin | 23.60 | 21.70-25.60 | Beta(221.64, 717.54) | [5] |
| PCSK9i + Statin | 57.93 | 54.91-60.95 | Beta(199.13, 144.52) | [6] |
| ASCVD Risk Reduction per 1 mmol/L LDL-C (%) | 21.00 | 19.0-23.0 | Beta(162.74, 611.81) | [7] |
| Adverse Event Management (Annual, %) |  |  |  | [8] |
| Statin-associated diabetes | 0.30 | 0.26-0.35 | Beta(90.6, 211.4) |  |
| Statin-associated myopathy | 0.24 | 0.20-0.28 | Beta(106.3, 336.4) |  |
| Medication Adherence (%) |  |  |  |  |
| Standard care | 50 | 41-69 | Beta(11.9, 11.9) | [9] |
| Electronic pillbox monitoring | 80 | 74-83 | Beta(123.5, 30.9) | [10] |
|  |  |  |  |  |
| *Disease Incidence & Mortality* |  |  |  |  |
| CVD Incidence (per 1000 PYs) |  |  |  |  |
| First event, Low risk | 4.4 | 3.2-6.0 | Gamma(19.36, 0.000227) | [1] |
| First event, medium risk | 11.5 | 9.2-14.3 | Gamma(33.06, 0.000348) |  |
| First event, high risk | 17.5 | 14.3-27.6 | Gamma(13.22, 0.001324) |  |
| Recurrent event (per 100 PYs) | 13.80 | 8.4-22.4 | Gamma(11.90, 0.01160) | [11] |
| CVD Mortality (per 100 PYs) |  |  |  |  |
| Post-first event | 11.80 | 6.4-17.2 | Beta(16, 120) | [1] |
| Hazard Ratio (Recurrent vs. First) | 2.00 | 1.0-3.0 | … | [12] |
|  |  |  |  |  |
| *Characteristics of HIV progress* |  |  |  |  |
| Progress of HIV/AIDS on ART, monthly |  |  |  |  |
| Stable on HIV | 0.954 | 0.931-0.99 | Beta (95.4, 4.6) | [13] |
| AIDS-related mortality (per 100 PYs) |  |  |  | [14] |
| Male | 3.55 | 3.52-3.63 | Gamma(1188.2, 334.7) |  |
| Female | 1.02 | 1.01-1.04 | Gamma(1040.4, 1020.0) |  |
|  |  |  |  |  |
| *Costs (USD)* |  |  |  |  |
| HIV/AIDS treatment (Annual) |  |  |  |  |
| ART treatment | 727.1 | 508.97-945.23 | Gamma (30, 24.237) | [15] |
| AIDS opportunistic infection | 1160.63 | 812.44-1508.82 | Gamma (69.37, 0.02) | ^[16]^ |
| Screening & Monitoring (One-time) |  |  |  |  |
| Lipid testing | 18.73 | 15.92-21.54 | Gamma(105.3, 5.624) | [17] |
| Risk assessment | 6.39 | 5.43-7.34 | Gamma(87.0, 13.62) | [18] |
| Risk registration | 59.69 | 41.78-77.60 | Gamma(11.11, 0.186) | [2] |
| Electronic pillbox intervention (One-time) | 51.67 | 25.84-77.51 | Triangular(25.84, 51.67, 77.51) | [19] |
| Platform fees | 3.59 | 1.80-5.39 | Triangular(1.80, 3.59, 5.39) | [20] |
| Technical support | 16.41 | 8.21-24.62 | Triangular(8.21, 16.41, 24.62) |  |
| Clinical monitoring | 3.42 | 1.71-5.13 | Triangular(1.71, 3.42, 5.13) |  |
| Medication (Monthly) |  |  |  |  |
| Pitavastatin 4mg | 8.80 | 6.38-23.87 | Gamma(1.85, 0.210) | [21] |
| Ezetimibe + Statin | 53.43 | 48.51-58.44 | Gamma(115.6, 2.164) |  |
| PCSK9i + Statin | 147.43 | 133.1-161.85 | Gamma(89.3, 0.606) |  |
| Adverse Event Management (Annual) |  |  |  | [22] |
| Statin-associated diabetes | 1139.75 | 968.79-1310.72 | Gamma(106.6, 0.0935) |  |
| Statin-associated myopathy | 1825.42 | 1551.60-2099.23 | Gamma(106.9, 0.05855) |  |
| CVD Event Management |  |  |  |  |
| First MACE treatment | 2873.47 | 1871.68-4566.65 | Gamma(5.82, 0.00203) | [23] |
| Post-first MACE management | 860.25 | 594.79-1125.66 | Gamma(50.4, 0.0586) |  |
| Recurrent MACE treatment | 4602.26 | 634.09-15004.06 | Log-Normal(8.434, 1.80) | [24] |
| Post-recurrent MACE management | 2786.41 | 2507.31-3064.37 | Gamma(156.2, 0.0561) | [25] |
|  |  |  |  |  |
| *Utility Weights* |  |  |  |  |
| No CVD with HIV infection | 0.965 | 0.88-0.97 | Beta (143.00, 10.11) | [26] |
| No CVD with AIDS | 0.785 | 0.7-0.87 | Beta (47.1, 12.9) |  |
| MACE with HIV | 0.285 | 0.256-0.314 | Beta (380.6, 955.3) | [27] |
| Post-first MACE with HIV | 0.756 | 0.680-0.832 | Beta (37.5, 12.1) |  |
| Post-recurrent MACE with HIV | 0.748 | 0.673-0.823 | Beta (37.4, 12.6) |  |
| MACE with AIDS | 0.238 | 0.214-0.262 | Beta (382.2, 1221.7) | [28] |
| Post-first MACE with AIDS | 0.631 | 0.568-0.694 | Beta (37.6, 22.0) |  |
| Post-recurrent MACE with AIDS | 0.625 | 0.562-0.688 | Beta (37.6, 22.5) |  |
| Death | 0 | … | … |  |
|  |  |  |  |  |
| *Other Economic Parameters* |  |  |  |  |
| Discount rate | 0.03 | 0-0.05 | Beta (8.14, 272.45) | [29] |
| Willingness to pay (USD) | 40335 | … | … | [30] |

The state-transition model operates using a monthly cycle length. All annual event rates cited from the literature (e.g., CVD incidence, recurrence, and mortality rates) were converted into monthly transition probabilities for use within the Markov model. The conversion was performed based on the assumption of a constant exponential risk, using the standard formula:

$$p_{monthly}=1-(1-p_{annual})^{\frac{1}{12}}$$

$p_{annual}$ is the annual probability derived from the cited rate per person-year. All transition probabilities used in the state-transition diagrams are expressed on a per-cycle (monthly) basis.

## 3.2 Dynamic Risk Factor Progression and Risk Calculation

To realistically simulate the evolution of an individual's CVD risk profile over time, the model incorporates annual progression rates for key risk factors, based on data from the *China Cardiovascular Health and Disease Report 2023* and other longitudinal studies. These parameters include the annual increase in systolic blood pressure and total cholesterol, the age- and sex-specific probability of developing diabetes, and the annual probability of smoking cessation (Table S3). In each model cycle, these rates are applied to update the simulated risk factors for each individual. The updated values are then used to recalculate the 10-year ASCVD risk using the China-PAR risk prediction equation, the coefficients of which are also provided (Table S4). This dynamic approach allows the model to capture the impact of the natural history of risk factors on CVD progression in the absence of intervention.

**Table S3 Annual Natural History Transition Probabilities and Risk Factor Progression Rates.**

| **Parameter** | **Base Case Value** | **Range** | **Source** |
| --- | --- | --- | --- |
| *Systolic Blood Pressure Increase (mmHg/year)* |  |  | China Cardiovascular Health and Disease Report 2023[31] |
| Male, 40-59 years | 0.5 | 0.40-0.60 |  |
| Female, 40-59 years | 0.4 | 0.30-0.50 |  |
| Male, ≥60 years | 0.3 | 0.20-0.40 |  |
| Female, ≥60 years | 0.2 | 0.10-0.30 |  |
|  |  |  |  |
| *Diabetes Incidence (%/year)* |  |  |  |
| Male, 40-44 years | 0.58 | 0.36-0.80 | National epidemiological survey data[32] |
| Male, 45-49 years | 0.32 | 0.13-0.50 |  |
| Male, 50-54 years | 0.19 | 0.02-0.37 |  |
| Male, 55-59 years | 0.14 | -0.04 to 0.32 |  |
| Male, 60-64 years | 0.11 | -0.07 to 0.30 |  |
| Male, 65-69 years | 0.13 | -0.05 to 0.31 |  |
| Male, 70-74 years | 0.04 | -0.16 to 0.24 |  |
| Male, 75-79 years | -0.39 | -0.66 to -0.12 |  |
| Male, 80-84 years | -1.07 | -1.46 to -0.67 |  |
| Female, 40-44 years | -0.39 | -0.66 to -0.12 |  |
| Female, 45-49 years | -0.62 | -0.83 to -0.41 |  |
| Female, 50-54 years | -0.38 | -0.56 to -0.20 |  |
| Female, 55-59 years | 0.08 | -0.09 to 0.25 |  |
| Female, 60-64 years | 0.32 | 0.14-0.49 |  |
| Female, 65-69 years | 0.5 | 0.30-0.70 |  |
| Female, 70-74 years | 0.55 | 0.30-0.79 |  |
| Female, 75-79 years | 0.04 | -0.30 to 0.38 |  |
| Female, 80-84 years | -0.66 | -1.18 to -0.14 |  |
|  |  |  |  |
| *Smoking Cessation Probability (%/year)* |  |  |  |
| 40-49 years | 0.6 | 0.2-1.5 | Population-based study  in Chinese adults[33] |
| 50-59 years | 0.7 | 0.3-1.8 |  |
| 60-69 years | 1 | 0.4-2.5 |  |
| 70-79 years | 1.2 | 0.5-3.0 |  |
| ≥80 years | 1.2 | 0.5-3.0 |  |

**Table S4 Parameters for the China-PAR Risk Prediction Equation^a^**

| **Parameter** | **Men** |  | **Women** |  |
| --- | --- | --- | --- | --- |
|  | **Coefficient** | **Coefficient × Value†** | **Coefficient** | **Coefficient × Value†** |
| Ln(age), y | 31.97 | 130.88 | 24.87 | 101.84 |
| Ln(treated SBP), mm Hg | 27.39 | - | 20.71 | - |
| Ln(untreated SBP), mm Hg | 26.15 | 127.28 | 19.98 | 97.26 |
| Ln(total cholesterol), mg/dL | 0.62 | 3.32 | 0.06 | 0.31 |
| Ln(HDL-C), mg/dL | -0.69 | -2.78 | -0.22 | -0.87 |
| Ln(waist circumference), cm | -0.71 | -3.12 | 1.48 | 6.46 |
| Current smoker (1=Yes, 0=No) | 3.96 | 0 | 0.49 | 0 |
| Diabetes (1=Yes, 0=No) | 0.36 | 0.36 | 0.57 | 0 |
| Geographic region (1=Northern China, 0=Southern China) | 0.48 | 0.48 | 0.54 | 0.54 |
| Urbanization (1=Urban, 0=Rural) | -0.16 | -0.16 | N/A | N/A |
| Family history of ASCVD (1=Yes, 0=No) | 6.22 | 0 | N/A | N/A |
| Ln(age)*Ln(treated SBP) | -6.02 | - | -4.53 | - |
| Ln(age)*Ln(untreated SBP) | -5.73 | -114.21 | -4.36 | -86.9 |
| Ln(age)*Current smoker | -0.94 | 0 | N/A | N/A |
| Ln(age)*Family history of ASCVD | -1.53 | 0 | N/A | N/A |
| Individual sum | N/A | 142.04 | N/A | 119.22 |
| Mean (coefficient × value) | N/A | 140.68 | N/A | 117.26 |
| Baseline survival | N/A | 0.97 | N/A | 0.99 |
| Estimated 10-year risk (%) | N/A | 11 | N/A | 10.1 |

^a^The complete equation and calculation methodology are as described in the China-PAR project.[34] N/A indicates the parameter is not included in the risk equation for that sex.

The model employs a fully dynamic method for calculating the monthly probability of a first-onset ASCVD event. In each annual cycle, the China-PAR risk prediction equation (see Table S4 for coefficients) is used to calculate a 10-year ASCVD risk for each individual based on their current, updated risk factor profile (e.g., age, systolic blood pressure, total cholesterol, smoking status). This 10-year risk is then converted to a monthly transition probability to align with the model's monthly cycle structure, using the standard formula under a constant hazard assumption:

Monthly Transition Probability = 1 - (1 - R₁₀)^(1/120)

where R₁₀ represents the 10-year ASCVD risk probability. This approach ensures that the model captures the continuous increase in an individual's risk as they age and their risk factors worsen, providing a more realistic simulation than using a static age-sex lookup table.

References

1.	Grinspoon, S.K., et al., Pitavastatin to Prevent Cardiovascular Disease in HIV Infection. N Engl J Med **2023**; 389(8): p. 687-699.

2.	Lei Bi, *[Lipid epidemiological characteristics, current status of statin application and simulation of the burden of lipid-lowering therapy in the Chinese population]*. 2022, Peking Union Medical College.

3.	Li, X., et al., Risk Assessment of Cardiovascular Disease in HIV Infected Individuals Receiving Antiretroviral Therapy - Shanghai Municipality, China, 2023. China CDC Wkly **2025**; 7(8): p. 271-276.

4.	Aberg, J.A., et al., Pitavastatin versus pravastatin in adults with HIV-1 infection and dyslipidaemia (INTREPID): 12 week and 52 week results of a phase 4, multicentre, randomised, double-blind, superiority trial. Lancet HIV **2017**; 4(7): p. e284-e294.

5.	Mikhailidis, D.P., et al., Meta-analysis of the cholesterol-lowering effect of ezetimibe added to ongoing statin therapy. Curr Med Res Opin **2007**; 23(8): p. 2009-26.

6.	Navarese, E.P., et al., Effects of Proprotein Convertase Subtilisin/Kexin Type 9 Antibodies in Adults With Hypercholesterolemia: A Systematic Review and Meta-analysis. Ann Intern Med **2015**; 163(1): p. 40-51.

7.	Mihaylova, B., et al., The effects of lowering LDL cholesterol with statin therapy in people at low risk of vascular disease: meta-analysis of individual data from 27 randomised trials. Lancet **2012**; 380(9841): p. 581-90.

8.	HPS2-THRIVE randomized placebo-controlled trial in 25 673 high-risk patients of ER niacin/laropiprant: trial design, pre-specified muscle and liver outcomes, and reasons for stopping study treatment. Eur Heart J **2013**; 34(17): p. 1279-91.

9.	Hope, H.F., et al., Systematic review of the predictors of statin adherence for the primary prevention of cardiovascular disease. PLoS One **2019**; 14(1): p. e0201196.

10.	Wei, X., et al., Effectiveness of a comprehensive package based on electronic medication monitors at improving treatment outcomes among tuberculosis patients in Tibet: a multicentre randomised controlled trial. The Lancet **2024**; 403(10430): p. 913-923.

11.	Haji, M., et al., Clinical Outcomes After Acute Coronary Syndromes or Revascularization Among People Living With HIV: A Systematic Review and Meta-Analysis. JAMA Netw Open **2024**; 7(5): p. e2411159.

12.	Boettiger, D.C., et al., Cost-effectiveness of statins for primary prevention of atherosclerotic cardiovascular disease among people living with HIV in the United States. J Int AIDS Soc **2021**; 24(3): p. e25690.

13.	Nosyk, B., et al., HIV-1 disease progression during highly active antiretroviral therapy: an application using population-level data in British Columbia: 1996-2011. J Acquir Immune Defic Syndr **2013**; 63(5): p. 653-9.

14.	Zhihui, D., et al., Trends in Mortality and Prevalence of Reported HIV/AIDS Cases — China, 2002–2021. China CDC Weekly **2023**; 5: p. 943.

15.	Lahong J, G.Z., Peng X, Qianqian X, Ji Z, Fan L., Unit cost of antiretroviral treatment of HIV/AIDS cases. Chin J Public Health **2013**; 29(11): p. 1580-3.

16.	Z;, Y., *Economic evaluation of PrEP prevention strategy in MSM population [Master]*. 2018, Chongqing Medical University.

17.	administration, S.H.S. *[Notice on adjusting the price of some medical services in this municipality]*. 2024.

18.	Wang, R., et al., Cohort Profile: ChinaHEART (Health Evaluation And risk Reduction through nationwide Teamwork) Cohort. Int J Epidemiol **2023**; 52(5): p. e273-e282.

19.	Southern Medical University. *An Implementation Study of Electronic Drug Boxes for Improving Medication Adherence in Tuberculosis Patients [DB/OL]*. 2025.

20.	Sweeney, S., et al., Unit costs and cost-effectiveness of a device to improve TB treatment adherence in China. IJTLD Open **2024**; 1(7): p. 299-305.

21.	Xiang, Y., et al., Cost-effectiveness of adding ezetimibe and/or PCSK9 inhibitors to high-dose statins for secondary prevention of cardiovascular disease in Chinese adults. Int J Technol Assess Health Care **2023**; 39(1): p. e53.

22.	Commission, N.H., *[China Health Statistics Yearbook 2022]*. 2022, Beijing: China Union Medical University Press.

23.	Li C., J.W., Economic burden among cardiovascular patients of middle-aged and elderly: an empirical analysis based on China Health and Retirement Longitudinal Survey data. Chin J Health Policy **2017**; 10(5): p. 75–80.

24.	Yang, H., et al., Cost-Effectiveness Analysis of Ezetimibe as the Add-on Treatment to Moderate-Dose Rosuvastatin versus High-Dose Rosuvastatin in the Secondary Prevention of Cardiovascular Diseases in China: A Markov Model Analysis. Drug Des Devel Ther **2020**; 14: p. 157-165.

25.	Li Y, L.D., Cost-effectiveness analysis of invasive versus conservative treatment in patients with acute non-ST-segment elevation myocardial infarction. China J Pharma Econ **2016**; 11(006): p. 9-13.

26.	Schneider, K., R.T. Gray, and D.P. Wilson, A cost-effectiveness analysis of HIV preexposure prophylaxis for men who have sex with men in Australia. Clin Infect Dis **2014**; 58(7): p. 1027-34.

27.	Feng, T., et al., Health economics assessment of statin therapy initiation thresholds for atherosclerosis prevention in China: a cost-effectiveness analysis. Int J Equity Health **2025**; 24(1): p. 31.

28.	Tan, Z., et al., Health-related quality of life as measured with EQ-5D among populations with and without specific chronic conditions: a population-based survey in Shaanxi Province, China. PLoS One **2013**; 8(7): p. e65958.

29.	WHO, *WHO guide to cost-effectiveness analysis*. Geneva: World Health Organization.

30.	National Bureau of Statistics of China, *Statistical Bulletin on National Economic and Social Development of the People's Republic of China for 2024*. 2025.

31.	National Center for Cardiovascular Diseases, *Report on Cardiovascular Health and Diseases in China 2023*. 2023, Beijing, China: Peking Union Medical College Press.

32.	Zhang, H., et al., Incidence, prevalence, and burden of type 2 diabetes in China: Trend and projection from 1990 to 2050. Chin Med J (Engl) **2025**; 138(12): p. 1447-1455.

33.	Zeng, Q., et al., Prevalence, cessation, and geographical variation of smoking among middle-aged and elderly adults in China: A population-based study. Tob Induc Dis **2024**; 22.

34.	Yang, X., et al., Predicting the 10-Year Risks of Atherosclerotic Cardiovascular Disease in Chinese Population: The China-PAR Project (Prediction for ASCVD Risk in China). Circulation **2016**; 134(19): p. 1430-1440.
